# Patients with first versus multiple episodes of self-harm: how do their profiles differ?

**DOI:** 10.1186/s12991-021-00351-5

**Published:** 2021-05-13

**Authors:** Philippe Golay, Louise Ostertag, Alessandra Costanza, Bénédicte Van der Vaeren, Yves Dorogi, Stéphane Saillant, Laurent Michaud

**Affiliations:** 1 Community Psychiatry Service, Department of Psychiatry, Lausanne University Hospital and University of Lausanne, Lausanne, Switzerland; 2 General Psychiatry Service, Treatment and Early Intervention in Psychosis Program (TIPP–Lausanne), Lausanne University Hospital and University of Lausanne, Lausanne, Switzerland; 3 Institute of Psychology, Faculty of Social and Political Science, University of Lausanne, Lausanne, Switzerland; 4 Liaison Psychiatry Service, Department of Psychiatry, Lausanne University Hospital and University of Lausanne, Lausanne, Switzerland; 5 Department of Psychiatry, Faculty of Medicine, University of Geneva (UNIGE), Geneva, Switzerland; 6 Pole of Psychiatry and Psychotherapy, Liaison Psychiatry Service, Hospital Centre of Valais Romand, Sion, Switzerland; 7 Center for Psychiatric Emergencies and Liaison Psychiatry, Department of General and Liaison Psychiatry, Neuchâtel Psychiatry Center, Neuchâtel, Switzerland

**Keywords:** Suicide, Self-harm, Multiple episodes, Risk factors, Repeaters

## Abstract

**Background:**

Self-harm (SH) is among the strongest predictors of further episodes of SH, suicide attempt, and death by suicide. People who repeteadly harm themselves are at even higher risk for suicide. Factors influencing the repetition are important to identify when assessing suicidal risk and thereafter to offer specific interventions. Therefore, this study aimed to compare first versus multiple episodes characteristics in a large sample of patients in french-speaking Switzerland.

**Method:**

We used the database from the French-speaking Swiss program for monitoring SH. Data of the psychiatric assessment of all adults admitted for SH were collected in the emergency department of four Swiss city hospitals between December 2016 and October 2019.

**Results:**

1730 episodes of SH were included. Several variables were significantly associated with multiple episodes, including diagnosis (over representation of personality disorders and under representation of anxiety disorders), professional activity (Invalidity insurance more frequent) and prior psychiatry care.

**Conclusions:**

Patients suffering from a personality disorder and those with invalidity insurance are at risk for multiple episodes of SH and should be targeted with specific interventions.

## Introduction

Together with suicide attempt (SA), self-harm^1^ (SH) [1, 2] is one of the strongest predictors of further episodes of SA, SH and completed suicide [3–7]. Moreover, in themselves, SA and SH lead to costly hospitalization [8], stigma [9], and difficulties in asking for help [10]. Among persons who self-harm, those with multiple episodes are at higher risk of dying by suicide [11] and, thus, represent an important target for prevention [12]. Previous research sought to find differences between those who engage in a single episode of self-harm versus repeated episodes. Identifying factors influencing the repetition is important to include this information in the suicidal risk assessment and then to offer specific interventions targeting modifiable risk factors. Moreover, this can help to improve the care of patients who harm themselves.

A systematic review in 2013 showed that unemployment, unmarried status, diagnosis of mental disorders, suicidal ideation (SI), stressful life events, and family history of suicidal behavior were associated with repetition of SA in adults [12]. In young people, another systematic review identified any personality disorder and any mood disorder as modifiable risk factors, and severity of hopelessness, SI and previous sexual abuse as associated with repetition of SH [13]. Recent studies on adults with a prospective or cross-sectional design also found several risk factors for SA or SH repetition. They included any mental disorder, impulsivity, borderline personality disorder, PTSD, substance misuse, severity of psychopathology, lethality of SA, high SI, unmarried status, living alone, younger age, low social support, no occupation, previous psychiatric treatment, history of sucide or major depression in the family, hopelessness and physical illness [14–22]. Finally, childhood maltreatment and/or sexual abuse have been associated with suicidal behaviors in a systematic review [23] and with repetition in two prospective studies [24, 25].

These heteogeneous results may in part be explained by low statistical power, most of the studies including between 60 and 300 patients. Moreover, they may reflect the unconsistency of definitions of multiple suicide attempters/patients with multiple SH episodes and the fact that people with a first episode at one point may further become repeaters (e.g., people who repeatedly harm themselves) [12]. Finally, they are certainly also related to wide differences in repetition patterns depending on location and cultural contexts [26], a recent systematic review namely highlighting important geographical differences in repetition of fatal and non-fatal SH [27]. Studies on specific regions are, thus, necessary. We could not identify any study on repetition of SH in Switzerland and aimed to compare first versus multiple episodes characteristics in a large sample of patients in French-speaking Switzerland. Following the existing literature, we hypothesized that specific socio-demographic and/or clinical factors would be independantly associated with repetition in our sample. Among the investigated factors were variables related to age, gender, social and professional status, lifestyle but also physical and mental health and detailed characteristics of the SH episode.

## Materials and methods

### The French-speaking Swiss program for monitoring SH

For this study, we used the database from the French-speaking Swiss program for monitoring SH. This monitoring has been described in full details elsewhere [28]. Briefly, it aimed to collect systematically data during the psychiatric assessment of all patients admitted for SH in four emergency departments (ED) of Swiss general hospitals between December 2016 and November 2019.

### Participants

All patients 18 years of age or older and admitted for SH in the four ED were included in this study (inclusion criteria). Patients under 18 were exluded (exclusion criteria). Patients who appeared multiple times in the database and patients who reported having made previous episodes of SH were included in the multiple episodes group. Data of the last episode were used in this study. Patients who declared no prior episode were included in the first episode group. Patients who apperead once in the database but without information on previous episodes were excluded. 1730 participants (mean age = 38.2; SD = 15.2) were included.

### Procedure

The data were based on information gathered through clinical evaluation by psychiatric residents [28]. Data were recorded through a paper form filled-in by the resident assessing the patient. The paper form [28] included items on socio-demographic characteristics (e.g., age, gender, nationality, problematic socioeconomic situation, migration in the past 10 years, civil status, invalidity insurance (pension for people who have been unable to work for health reasons into the working world.)) and clinical information (e.g., first International Classification of Diseases diagnosis (ICD-10) coded by sections (see Table 2), past history of self-harm, existing psychiatric illnesses, psychiatric history, existing follow-up) and detailed information on the patient's suicidal process (e.g., suicidal intent, method of self-harm and severity of the self-harm episode, protective and precipitating factors). Psychiatric diagnoses were recorded according to the ICD-10 under the supervision of senior psychiatrists; collectors could mention up to three diagnoses by order of importance [28]. Name, surname, gender and birth date were merged into one string and subjected to the Message Digest 5 algorithm (MD5) which creates a 128-bit cryptographic hash. This unique text string allowed us to ensure patient anonymity in the database while allowing us to identify participants with multiple episodes within one site or from one site to another.

### Statistical analysis

Comparisons between groups were performed with independent t-tests for continuous variables and Pearson's Chi-Square tests (or Fisher Exact tests with exact or Monte-Carlo estimation when needed) for categorical variables. To highlight the most important variables independent of each other, a multivariate logistic model was estimated. Multiple Imputation was deemed not feasible given the very large proportion of nominal variables. Only variables with less than 15% of missing data and reaching a *p* < 0.05 level of significance when comparing the two groups were included as independent variables. All statistical analyses were performed with IBM-SPSS 26. All statistical tests were two tailed and significance was determined at the 0.05 level.

## Results

Comparison of the socio-demographic variables (Table 1) showed that females (*p* = 0.005) and Swiss nationals (*p* = 0.014) were overrepresented in the multiple episodes group. Problematic socioeconomic situation (*p* < 0.001), living single lifestyle (*p* < 0.001), and single civil status (*p* < 0.001) were also more likely in the multiple episodes group. Examination of level of education revealed that patients of the multiple episodes group were also more likely to only have basic/elementary training (*p* < 0.001). Patients who made multiple episodes were more likely to be working part time or to benefit from the invalidity insurance (*p* < 0.001). They were also more likely to have another legal representative than themselves (*p* < 0.001). Considering clinical variables (Table 2), patients who made multiple episodes were less likely to have a diagnosis of anxiety/stress-related (F 4) disorder and more likely to have a diagnosis of personality disorder (*p* < 0.001). They were more likely to suffer from physical pain (*p* = 0.010) and/or physical illnesses (*p* = 0.040). Location at the time of SH was slightly less likely to be at home for patients with multiple episodes (*p* = 0.006). Patients of the multiple episodes group were less likely to arrive at the emergency departments with family or friends and more likely to arrive alone (*p* = 0.043). They were more often intoxicated at the time of the episode (*p* < 0.001) and use of any substance during the last 3 months was higher (*p*-values ranging between < 0.001 and 0.012). Considering existing follow-up at time of SH, patients with multiple episodes were less likely to have no follow-up and more likely to have psychiatric care (*p* < 0.001). Post-self-harm follow-up was less likely to be outpatient public psychiatry network and more likely to be a voluntary or involuntary psychiatric hospitalization (*p* < 0.001). Finally, significant events related to work situation (*p* = 0.004) or harassment/mobbing (*p* = 0.032) were less frequent for patients who had repeated episodes of SH.

**Table 1:** Socio-demographic variables: comparison between patients with first versus multiple episodes of SH (*N* = 1730)

	Patients with first episode *N = *764 (44.2%)	Patients with multiple episodes *N* = 966 (55.8%)	Statistics	*P*-value
Sites, % (*N*)			χ^2^(3) = 4.123	.249
Lausanne	48.6 (371)	50.8 (491)		
Geneva	11.8 (90)	13.7 (132)		
Neuchatel	19.6 (150)	16.6 (160)		
Valais	20.0 (153)	18.9 (183)		
Gender, % (*N*)			Fisher’s exact test	.005
Male	47.1 (360)	40.2 (388)		
Female	52.9 (404)	59.7 (577)		
Other	0.0 (0)	0.1 (1)		
Age, Mean (SD)	38.74 (16.41)	37.82 (14.07)	t(1506.150) = 1.235	.217
Legal status, % (*N*)			Fisher’s exact test	.014
Swiss nationality	67.2 (399)	74.5 (545)		
Legally transiting in Switzerland	1.5 (9)	0.7 (5)		
Permit B	8.1 (48)	5.1 (37)		
Permit C	10.6 (63)	8.1 (59)		
Permit F	2.0 (12)	1.1 (8)		
Permit L	0.3 (2)	0.8 (6)		
Permit N	1.5 (9)	1.6 (12)		
NEM status	2.4 (14)	1.5 (11)		
Clandestine	4.4 (26)	4.0 (29)		
Non-accompanied minor	0.5 (3)	0.0 (0)		
Other	1.5 (9)	2.7 (20)		
Problematic socioeconomic situation, % (*N*)	51.9 (356)	63.3 (519)	χ^2^(1) = 19.932	< .001
Lifestyle, % (*N*)			χ^2^(8) = 34.781	< .001
By him/herself	21.8 (165)	28.2 (271)		
Couple without children	19.4 (147)	14.2 (136)		
Couple with children	24.9 (189)	20.0 (192)		
By his or her parents	16.4 (124)	14.2 (136)		
Shared accommodation	4.5 (34)	3.8 (36)		
Foster care, institution for the elderly, etc.	4.1 (31)	7.7 (74)		
Incarcerated	4.6 (35)	6.3 (60)		
Homeless	1.8 (14)	1.7 (16)		
Other	2.5 (19)	4.1 (39)		
Civil status, % (*N*)			χ^2^(4) = 19.433	< .001
Single	49.0 (365)	54.3 (503)		
Married or registered partnership	29.4 (219)	20.3 (188)		
Divorced	12.8 (95)	16.0 (148)		
Separated	6.3 (47)	6.7 (62)		
Widowed	2.6 (19)	2.8 (26)		
Level of education, % (*N*)			Fisher’s exact test	< .001
Compulsory schooling	20.9 (101)	26.7 (141)		
Apprenticeship	32.6 (158)	30.5 (161)		
Maturity diploma	6.8 (33)	7.6 (40)		
Professional/commercial/technical school	17.8 (86)	11.7 (62)		
University	16.3 (79)	14.4 (76)		
No completed schooling	2.5 (12)	3.0 (16)		
Out of school	1.7 (8)	2.5 (13)		
Specialized courses	0.8 (4)	2.5 (13)		
Other	0.6 (3)	1.1 (6)		
Professional activity, % (*N*)			χ^2^(7) = 86.099	< .001
Apprentice	15.9 (114)	13.0 (115)		
Full-time worker	23.3 (167)	14.7 (130)		
Part-time worker	9.3 (67)	7.1 (63)		
Household activity	2.4 (17)	2.7 (24)		
Unemployed	27.6 (198)	29.2 (259)		
Retired or equivalent	7.4 (53)	3.6 (32)		
Invalidity insurance	8.9 (64)	24.4 (216)		
Other	5.3 (38)	5.4 (48)		
Legal representative, % (*N*)			Fisher’s exact test	< .001
Him/herself	96.2 (713)	87.6 (781)		
Parents	1.1 (8)	2.0 (18)		
Curatorship	2.3 (17)	9.8 (87)		
Other	0.4 (3)	0.7 (6)		

For multiple SH, the data of the last episode were used

*IQR* s Inter-quartile Range

^a^Patients with multiple episodes > Patients with first episode

**Table 2:** Clinical variables: comparison between patients with first versus multiple episodes of SH (*N = *1730)

	Patients with first episode *N* = 764 (44.2%)	Patients with multiple episodes *N* = 966 (55.8%)	Statistics	*P*-value
Diagnostic, % (*N*)			Fisher’s exact test	< .001
Dementia F0	0.0 (0)	0.8 (7)		
Alcohol use F10	5.8 (40)	4.8 (44)		
Substance use F11–F19	2.3 (16)	1.8 (17)		
Schizophrenia F2	3.9 (27)	7.8 (72)		
Bipolar disorders F3-M	1.6 (11)	2.4 (22)		
Depression F3-D	34.0 (236)	32.7 (301)		
Anxiety/stress-related disorders F4	39.7 (276)	17.3 (159)		
Behavioral syndromes assoc. w. physiological disturbances F7-F9	1.6 (11)	2.1 (19)		
Personality disorder F6	11.2 (78)	30.4 (280)		
Physical pain, % (*N*)	20.1 (145)	25.6 (223)	χ^2^(1) = 6.705	.010
Disabling physical illness, % (*N*)	13.8 (97)	17.7 (150)	χ^2^(1) = 4.206	.040
Method of self-harm, % (*N*)			Fisher’s exact test	.065
Self-poisoning (medication)	59.1 (450)	59.2 (571)		
Self-poisoning (other substance)	4.6 (35)	3.2 (31)		
Cutting	12.3 (94)	14.0 (135)		
Firearm	0.3 (2)	0.2 (2)		
Jumping from a height	7.7 (59)	6.0 (58)		
Hanging or asphyxiation	6.4 (49)	7.3 (70)		
Drowning	2.0 (15)	0.8 (8)		
Jumping/lying in front of a moving object	2.2 (17)	2.8 (27)		
Multiple methods	2.5 (19)	3.9 (38)		
Other	0.3 (2)	0.5 (5)		
Burning and immolation	1.3 (10)	0.7 (7)		
Physical auto-aggressiveness	1.2 (9)	0.6 (6)		
Ingestion of a foreign object	0.1 (1)	0.7 (7)		
Location at the time of the self-harm episode, % (*N*)			χ^2^(5) = 16.512	.006
Home	74.6 (565)	71.5 (686)		
Workplace/school	1.6 (12)	0.5 (5)		
Medical/social institution, prison	7.4 (56)	10.8 (104)		
Public space	9.2 (70)	10.8 (104)		
Isolated place	3.6 (27)	2.0 (19)		
Other	3.6 (27)	4.4 (42)		
Level of suicidal intent, % (*N*)			χ^2^(2) = 3.813	.149
Clear suicidal intent	48.9 (372)	52.8 (504)		
Unclear suicidal intent	26.8 (204)	26.7 (255)		
No suicidal intent	24.2 (184)	20.5 (196)		
Notable Seriousness of the episode, % (*N*)	17.6 (132)	18.1 (170)	χ^2^(1) = 0.067	.796
Arrival at the ER, % (*N*)			χ^2^(5) = 11.485	.043
Alone, on its own initiative	10.1 (75)	14.4 (132)		
With family/friends, on their impulse	22.2 (164)	18.3 (168)		
By ambulance, called by the patient	5.4 (40)	5.4 (50)		
By ambulance, called by other	49.2 (364)	49.0 (450)		
With the police, called by the patient	0.8 (6)	1.6 (15)		
With the police, called by other	12.3 (91)	11.3 (104)		
Intoxication at the time of the episode, % (*N*)	35.7 (261)	44.9 (403)	χ^2^(1) = 14.186	< .001
Use of substance during the last 3 months, Median (IQR)				
Alcohol	2.0 (3.0)	2.0 (3.0)	U = 149,110.0	< .001^a^
Cannabis	1.0 (0.0)	1.0 (0.0)	U = 138,545.0	.003^a^
Unprescribed medicine	1.0 (0.0)	1.0 (0.0)	U = 102,208.0	< .001^a^
Cocaine	1.0 (0.0)	1.0 (0.0)	U = 155,606.5	.012^a^
Opiates	1.0 (0.0)	1.0 (0.0)	U = 141,418.5	< .001 ^a^
Pre-self-harm episode follow-up, % (*N*)			Fisher’s exact test	< .001
None	33.6 (249)	18.5 (172)		
General practitioner	20.8 (154)	12.0 (112)		
Outpatient public psychiatry network	15.1 (112)	31.0 (288)		
Psychologist or psychiatrist in private practice	25.8 (191)	32.8 (305)		
Other healthcare professional	1.9 (14)	1.2 (11)		
Voluntary psychiatric hospitalization	0.8 (6)	2.6 (24)		
Involuntary psychiatric hospitalization	0.5 (4)	0.8 (7)		
Psychiatric hospitalization, unspecified	0.3 (2)	0.3 (3)		
Non-psychiatric hospitalization (liaison)	0.9 (7)	0.5 (5)		
Social worker	0.1 (1)	0.3 (3)		
Post-self-harm episode follow-up, % (*N*)			Fisher’s exact test	< .001
None	4.1 (30)	2.9 (27)		
Outpatient public psychiatry network	34.4 (251)	25.3 (233)		
General practitioner	3.2 (23)	1.3 (12)		
Psychologist or psychiatrist in private practice	15.8 (115)	14.9 (137)		
Other healthcare professional	1.4 (10)	1.0 (9)		
Voluntary psychiatric hospitalization	22.4 (163)	32.3 (297)		
Involuntary psychiatric hospitalization	13.0 (95)	19.0 (175)		
Non-psychiatric hospitalization (liaison)	4.8 (35)	2.4 (22)		
Psychiatric hospitalization, unspecified	0.4 (3)	0.4 (4)		
Social worker	0.5 (4)	0.4 (4)		
Realization level of the self-harm episode, % *N*)			χ^2^(2) = 2.122	.346
Completed	67.2 (205)	72.2 (314)		
Interrupted	21.3 (65)	17.9 (78)		
Aborted	11.5 (35)	9.9 (43)		
Significant recent event, suffering related to work situation, % (*N*N)	16.2 (46)	10.8 (42)	χ^2^(1) = 4.212	.004
Significant recent event, harassment at work/mobbing, % (*N*)	4.3 (12)	1.6 (6)	χ^2^(1) = 4.599	.032

For multiple episodes of SH, the data of the last episode were used

*IQR* Inter-quartile Range

^a^Patients with multiple episodes > Patients with first episode;

Results of the multivariate logistic model showed that only three variables remained significant when all variables were considered altogether. Diagnosis (Anxiety/stress-related F4 versus Depression F3-D as the reference category, Odds ratio = 0.508, *p* < 0.001; Personality disorder F6 versus Depression F3-D as the reference category, Odds ratio = 2.010, *p* = . 002), Professional activity (Invalidity Insurance versus Working full time as the reference category, Odds ratio = 2.174, *p* = 0.009) and Pre-self-harm episode follow-up (Outpatient public psychiatry network versus None, Odds ratio = 2.421, *p* < 0.001).

## Discussion

Patients with multiple episodes of SH differed from those with a first episode on several variables. The most important ones were diagnosis (over representation of personality disorders and under representation of anxiety/stress-related disorders), professional activity (Invalidity insurance more frequent) and prior psychiatry care.

Regarding diagnosis, we found repeaters to suffer more frequently from a personality disorder. Our analyses did not differentiate between specific personality disorders but we had a high prevalence of the diagnosis of borderline personality disorder in our sample (62.01%; 222/358). It is, thus, likely that this result reflects a risk with borderline personality disorders, in line with the previous research showing borderline personality disorder or traits to be associated with repetition both in adults [15, 18, 22, 29] and in young people [13]. Persons with a borderline personality disorder should be offered specific treatment to reduce repetition, such as Dialectical Behavior Therapy [30], Mentalization-based treatment [31] or Transference-focused psychotherapy [32]. Interestingly, anxiety/stress-related disorder was found to be less frequent in repeaters, a result we did not find in previous studies. While the severity of psychopathology was found to be related to repetition [5, 22] and since an important proportion of our anxiety/stress-related disorders were adjustment disorders (75.63%; 329/435), we could suppose that adjustment disorder, a frequently used diagnosis [33], was more frequently made for patients with a less severe psychopathology. However, following the interpersonal theory of suicide [34, 35], this could also reflect a decrease in the anxiety level with the repetition of SH. Indeed, habituation and activation of adverse processes in response to repeated exposure to physically painful and/or fear-generating experiences reduce not only the fear of death but also physical anxiety.

Our results on occupation underline the importance of the social context for repetition of SH and is in line with previous findings on absence of occupation as a risk factor for repetition of SA [12, 21]. Since having an occupation is a major way to be and stay connected with people and to get support if needed, this result also echoes previous research showing that low social support is associated with repetition of SA [16, 19]. Furthermore, low social support is related to loneliness, which can increase interpersonal difficulties—both with relatives and with health care providers—in a vicious circle, and perceived burdensomeness—a risk factor for suicidal behavior [35, 36]—prevents repeaters from reporting their feelings and seeking help from peers and family. Clinicians should be aware of the specific issues related to interpersonal relationship, especially with patients suffering from a personality disorder [37]. The fact that, in unvariate analysis, patients with multiple episodes were more likely to present alone to the emergency departements may also be related to this low social support and this population requires special attention. At an individual level, when meeting suicidal patients, health professionals shoud consider social determinants as well as mental-health problems [38, 39]. Social difficulties should be targeted when elaborating a treatment plan: social workers should be included in the treatement and mobilization of social support has to be specifically adressed. At a population level, politics should be aware of the importance of having a job as a protective factor against repetition of SH, since SH also represents an important economic burden [8].

Interestingly, we found no difference between our two groups on the intent to die, recorded as absent, unclear or present. This differs from several studies [5, 19, 22] and a systematic review [12] showing repetition to be associated with the intensity of SI in suicide attempters. It may be that our group of multiple episodes include an important proportion of non-suicidal self-injury, thus mitigating this association.

Several other variables that were highlighted may deserve attention. While intoxication at the time of the SH and substance use were associated to repetition in univariate analysis, this did not remain significant in multivariate analysis. One study found men repeaters to use substance more frequently [20] and a study including veterans (probably mostly men) [18] identified substance use disorders as more frequent in repeaters. We did not perform a separate gender analysis but this negative result underscores the need of specific research on pattern of repetition across women and men. Finally and interestingly, age, realization of the self-harm episode, level of suicidal intent and seriousness of the episode did not differ between the two groups.

This study has several limitations. First, some variables had some non-ignorable amount of missing data and several significant variables were excluded from the multivariate regression model (recent significant events for work situation and harassment/mobbing, education and use of substance during the last three months). Second, it is likely that patients with a first episode of SH at one point may further become repeaters [12]. Third, this study is cross-sectional in design and longitudinal studies may be warranted to strengthen our findings.

## Conclusion

Repeated SH represents a high risk for suicidal patients and monitoring SH is an important yet difficult endeavor. In our study, patients suffering from a personality disorder were at risk for multiple episodes of SH. Regarding individual actions, clinicians should be especially vigilant about these patients and offer them specific and dedicated interventions. With this population, they should also be aware of their own emotional reactions, which may hinder proper assessment and treatment through adverse countertransference [38, 39].

Our study also showed that people with invalidity insurance were more prone to repeat SH; this highlights the importance of the social context for suicidal behaviors. Obviously, further studies are needed to determine to which extent this could be partly accounted for by variables like public stigma and self-stigma. Regarding community responsibilities and policy implication, additional regulations are needed to warrant patients’ access to specialized care and adequate treatment, all the more so after factors associated with repetition have been identified. To reach this goal, studies on specific interventions (e.g., sustained social work, vocational interventions, and psychotherapeutic interventions) to reduce repetition are warranted. Awareness campaigns towards health professional from various backgrounds must also be developed in such a way that the risk factors are known and investigated.

## Data Availability

The datasets generated and analyzed during the current study are not publicly available because public archiving of data was not explicitly authorized by the ethic committee. Nevertheless, anonymous data are available from the corresponding author on reasonable request.

## Footnotes

1 For this study, self-harm is defined as “all non-fatal intentional acts of self-poisoning or self-injury, irrespective of degree of suicidal intent or other types of motivation” [1], thus including both DSM 5 non-suicidal self-injury and other acts of self-harm with various suicidal intents, following a dimensional rather than categorical approach to the phenomenon [2].
